# Theory of current-driven skyrmions in disordered magnets

**DOI:** 10.1038/s41598-018-24693-5

**Published:** 2018-04-20

**Authors:** Wataru Koshibae, Naoto Nagaosa

**Affiliations:** 1 RIKEN Center for Emergent Matter Science (CEMS), Wako, Saitama 351-0198 Japan; 2 Department of Applied Physics, The University of Tokyo, 7-3-1, Hongo, Bunkyo-ku, Tokyo, 113-8656 Japan

## Abstract

An emergent topological particle in magnets, skyrmion, has several unique features distinct from the other magnetic textures such as domain wall, helical structure, and vortex. It is characterized by a topological integer called skyrmion number *N*_*sk*_, which counts how many times the directions of the magnetic moments wrap the unit sphere. This *N*_*sk*_ gives the chiral nature of the skyrmion dynamics, and leads to the extremely small critical current density *j*_*c*_ for the current-driven motion in terms of spin transfer torque effect. The finite *j*_*c*_ indicates the pinning effect due to the disorder such as impurities and defects, and the behaviors of skyrmions under disorder have not been explored well theoretically although it is always relevant in real systems. Here we reveal by a numerical simulation of Landau-Lifshitz-Gilbert equation that there are four different skyrmion phases with the strong disorder, i.e., (A) pinned state, (B) depinned state, (C) skyrmion multiplication/annihilation, and (D) segregation of skyrmions, as the current density increases, while only two phases (A) and (B) appear in the weak disorder case. The microscopic mechanisms of the new phases (C) and (D) are analyzed theoretically. These results offer a coherent understanding of the skyrmion dynamics under current with disorder.

## Introduction

Skyrmion in magnets is an emergent topological particle made from many spins swirling and pointing all the directions^1–21^. These magnetic skyrmions were first found by neutron scattering experiment as the triangular crystal form^7^, while later as individual particles in gas form as well as the crystal by Lorentz transmission electron microscopy^9^. Their motion is driven by the current^22,23^ through the spin transfer torque (STT) effect with the extremely small threshold current density ${j}_{c}\sim {10}^{6}$ A/m^2^ compared with that for the domain wall ~10^10^–10^12^ A/m^2^ ^24,25^. It is discussed that this high mobility comes from the chiral dynamics of the skyrmions, i.e., the velocity is perpendicular to the force acting on it, analogous to the charged particle under external magnetic field^26^. This makes a skyrmion to avoid an impurity, and reduces the pinning effect. Recently, room temperature skyrmions due to the interface Dzyalosinskii-Moriya interaction^27–29^ in multi-layered systems^30–34^ have been developed. In such systems, it is reported^32,34^ that the current driven skyrmions show pinned behavior and sometimes the skyrmion disappears by the current. The defects/impurities seriously influence the current driven skyrmion dynamics. The dynamics of driven skyrmions with disorder has been studied by numerical simulation based on the Thiele equation for each skyrmion, and three phases, i.e., pinned glass, moving liquid, and moving crystal, have been identified in the strong pinned case^35–38^. However, as the impurity density becomes high and the inter-impurity distance is shorter than the size of the skyrmion and impurity potential becomes strong, the deformation of skyrmions and spin wave emission occur. These phenomena cannot be captured by the Thiele equation for the center of mass motion which assumes the rigid shape of skyrmions. By comparing the results of Thiele equation^35–38^ with ours presented below, one can identify the effects of the internal deformation and spin wave emission, which lead to the different behaviors in the strong disorder case while both are consistent with each other in the weak disorder case.

**Figure 1:**
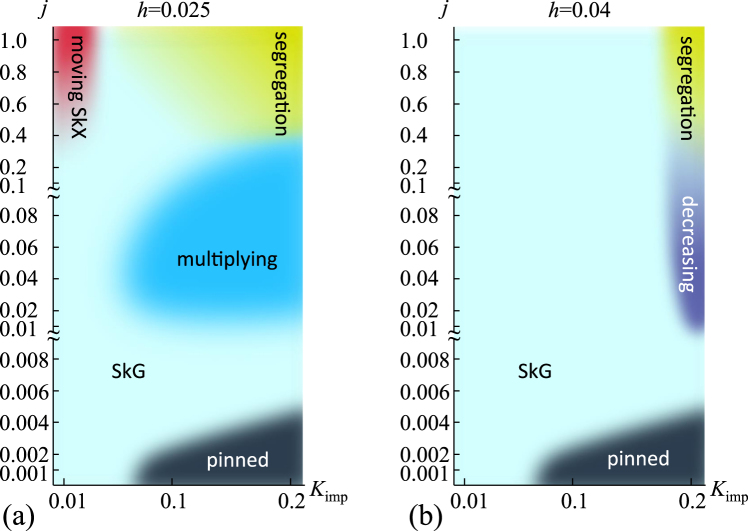
Skyrmion phases in disordered system. The external magnetic field *h* and the easy-axis anisotropy *K*_i*mp*_ at the impurity sites are measured in the energy unit of the exchange coupling *J*. The current density *j* is measured in the unit of 2*eγJ*/(*pa*^2^) which corresponds to $\sim {10}^{13}$ A/m^2^ in the three-dimensional bulk sample for the polarization of magnet *p* = 0.2 and the lattice constant *a* = 5Å. Left (right) panel is the result for *h* = 0.025 (*h* = 0.04) where SkX (F) phase is stabilized at *j* = 0. In the figures, SkG indicates the skyrmion gas state (see text).

In this paper, we investigate the current-driven dynamics of skyrmions with disorder by numerically solving the Landau-Lifshitz-Gilbert (LLG) equation for spins. We found four distinct phases as the current density is increased in the strongly disordered case, i.e., (A) pinnned state, (B) depinned state, (C) skyrmion multiplication/annihilation, and (D) segregation of skyrmions, while only two phases (A) and (B) appear in the weak disorder case. The microscopic processes in each phases are analyzed as shown in Fig. 1, as well as the spectroscopic information.

## Results

### Model and Simulation

We focus on the chiral magnet with Dzyaloshinskii-Moriya (DM) antisymmetric spin-orbit interaction^27–29^, whose model Hamiltonian on the two-dimensional square lattice is given as

1 $$\begin{array}{rcl} {\mathcal H}  & = & -\,J\sum _{{\boldsymbol{r}}}{{n}}_{{\boldsymbol{r}}}\cdot ({{n}}_{r{\boldsymbol{+}}\hat{{\boldsymbol{x}}}}+{{n}}_{{\boldsymbol{r}}+\hat{{\boldsymbol{y}}}})\\  &  & +\,D\sum _{{\boldsymbol{r}}}({{n}}_{{\boldsymbol{r}}}\times {{n}}_{{\boldsymbol{r}}+\hat{{\boldsymbol{x}}}}\cdot \hat{{\boldsymbol{x}}}+{{n}}_{{\boldsymbol{r}}}\times {{n}}_{{\boldsymbol{r}}+\hat{{\boldsymbol{y}}}}\cdot \hat{{\boldsymbol{y}}})\\  &  & -\,{K}_{{\rm{imp}}}\sum _{{{\bf{r}}}_{{\rm{i}}}\in {\rm{\Lambda }}}{({n}_{z,{{\boldsymbol{r}}}_{{\rm{i}}}})}^{2}-h\sum _{{\boldsymbol{r}}}{n}_{z,{\boldsymbol{r}}},\end{array}$$

with $\hat{{\boldsymbol{x}}}$ and $\hat{{\boldsymbol{y}}}$ being the unit vectors in the *x*- and *y*- directions, respectively. The lattice constant is taken as the unit of length. The first term represents the ferromagnetic interaction and the second one is the DM interaction stabilizing the Bloch winding magnetic texture. The easy-axis anisotropy *K*_imp_ is introduced to the random sites ***r***_i_ ∈ Λ (Λ: set of the random sites), which represents the disorder effect. The Zeeman effect by the external magnetic field *h* perpendicular to the *xy* plane is considered by the last term. Let us first discuss the magnetic states without the disorder, i.e., *K*_imp_ = 0. The competition between the ferromagnetic and the DM interactions results in the single-*q* helix state with *q* = *D*/*J*, for *h* = 0. Usually $D\ll J$, and therefore the period of the helix *ξ* = 2*π*/*q* = 2*π*(*J*/*D*) is much longer than the lattice constant, which justifies the continuum approximation. The skyrmion crystal state (SkX) appears in the intermediate *h* between the helix state for low *h* and the ferromagnetic state for high *h*^39,40^.

The LLG equation is given by:

2 $$\begin{array}{rcl}\frac{{\rm{d}}{{n}}_{{\boldsymbol{r}}}}{{\rm{d}}t} & = & -\,\gamma \frac{\partial  {\mathcal H} }{\partial {{n}}_{{\boldsymbol{r}}}}\times {{n}}_{{\boldsymbol{r}}}+\alpha {{n}}_{{\boldsymbol{r}}}\times \frac{{\rm{d}}{{n}}_{{\boldsymbol{r}}}}{{\rm{d}}t}\\  &  & -\,({j}\cdot \nabla ){{n}}_{{\boldsymbol{r}}}+\beta [{{n}}_{{\boldsymbol{r}}}\times ({j}\cdot \nabla ){{n}}_{{\boldsymbol{r}}}],\end{array}$$

where *α* is the Gilbert damping constant (see also Methods). The last two terms in Eq. (2) represent the STT effect due to the spin polarlized electric current density ***j*** with the coefficient of the non-adiabatic effect *β*. We use *J* for the unit of *h*, *D* and *K*_imp_, and 1/(*γJ*) for the unit of time *t*. (*p*: polarization of magnet). (See Methods for computational details). Typically $J\sim {10}^{-3}$ eV and the unit 1/(*γJ*) becomes ∼0.7 ps for ${\gamma }={g}_{s}{{\mu }}_{B}/\hslash$ (*g*_*s*_: electron spin *g*-factor, *μ*_*B*_: Bohr magneton). The unit of the electric current density *j* = |***j***| is 2*eγJ*/(*pa*^2^) and is typically $\sim 1.0\times {10}^{13}$ A/m^2^ for the polarization of magnet *p* = 0.2 and the lattice constant *a* = 5Å.

Figure 1 summarizes the dynamical phase diagrams of the current-driven skyrmions with disorder. In this study, we use *D*/*J* = 0.2 and introduce the impurity sites with 5% concentration and investigate the dynamics of skyrmions for various strength of impurity *K*_imp_. In this case, a skyrmion has a spatial extent $\sim \xi$. The effect of an impurity on the skyrmion discussed here is characterized by an impurity potential ${K}_{{\rm{imp}}}\mathrm{[1}-{({n}_{z,{{\bf{r}}}_{{\rm{i}}}})}^{2}]$ with the spatial extent *ξ* for the center of mass dynamics of the skyrmion. For 5% impurity concentration, because the averaged impurity-impurity distance is about 4 lattice spacings and is much smaller than $\xi \sim 30$ lattice spacings, the model discussed here is for the grain defects and not for point defects for the skyrmion dynamics.

We start with the results for *h* = 0.025 (see Fig. 1(a)). Here, we find the skyrmion pinned state, the skyrmion gas (SkG) state, the skyrmion multiplication behavior and the skyrmion segregation behavior. To investigate these states/behaviors in more detail, we first examine the single skyrmion dynamics in the disordered system.

### Single skyrmion dynamics: pinned, depinned and multiplication of skyrmion

**Figure 2:**
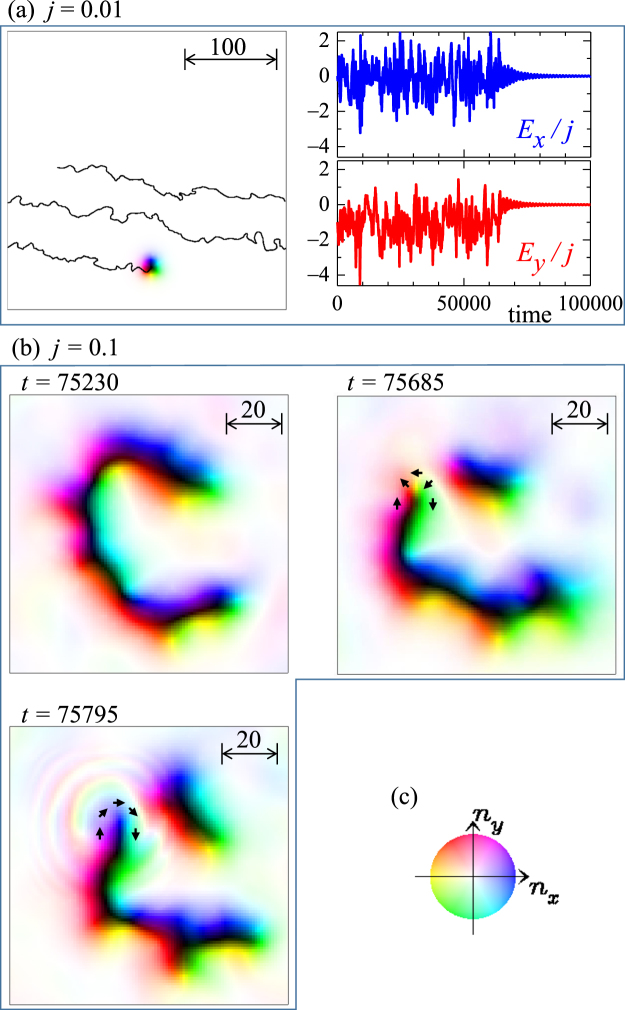
Single skyrmion dynamics in disordered system. (see text) A parameter set {*h* = 0.025, *K*_i*mp*_ = 0.2, *α* = *β* = 0.01} is used. (**a**) The skyrmion dynamics for *j* = 0.01. Left panel shows the magnetic texture at *t* = 100000 using color code (**c**) with trajectory by solid line. Right panels show the time dependence of *E*_*x*_/*j* (blue line) and *E*_*y*_/*j* (red line) (see text). (**b**) The skyrmion multiplication for *j* = 0.1. The magnetic textures at *t* = 75230, *t* = 75685 and *t* = 75795 are shown. (**c**) Color code for in-plane magnetic moment. Blue (yellow) means positive (negative) *n*_*x*_ direction. In the representation of the magnetic texture, the brightness means the out-of-plane magnetic moment ***n***, i.e., white (black) is for *n*_*z*_ = +1 (−1). (See also the supplementary movies).

In Fig. 2(a), the single skyrmion dynamics driven by ***j*** = (−*j*, 0) = (−0.01, 0) in the disordered system for {*h* = 0.025, *K*_imp_ = 0.2, *α* = *β* = 0.01} is shown. In the left panel of Fig. 2(a), the solid line represents the trajectory of the motion of the skyrmion in the periodic boundary condition. The skyrmion motion is disturbed by impurities and eventually the skyrmion is totally pinned. The final magnetic texture of the pinned skyrmion is shown in the left panel of Fig. 2(a). Under the condition *α* = *β*, in the absence of impurities *K*_imp_ = 0, the Hall angle of the current driven skyrmion motion is zero. In the disordered system, however, as discussed by the previous studies^35–38,41^, the skyrmion motion shows the Hall behavior during its motion as seen in the trajectory in Fig. 2(a), i.e., on the whole, the skyrmion is moving down in the panel. This is because of the skew dynamics of the skyrmion^1^: The *vorticity* of the skyrmion drives a dynamics to avoid the impurity similar to the vortices in superconductors^42,43^. Under the successive scattering by the impurities, the Hall behavior is brought about even in the case of *α* = *β* in contrast to the clean case^26^. Note that the Hall angle is *not* ~90 degree with respect the current direction even near the threshold current density different from what is expected from the analytic theory^26,44^.

To evaluate the velocity of the moving skyrmion, we define the quantity,

3 $${\boldsymbol{E}}=\frac{1}{2\pi }\iint {{\boldsymbol{e}}}_{{\boldsymbol{r}}}\,{d}^{2}r,$$

with the emergent e-field^44–46^

4 $${{\boldsymbol{e}}}_{{\boldsymbol{r}}}=\frac{1}{2}({{\boldsymbol{n}}}_{{\boldsymbol{r}}}\cdot {\partial }_{x}{{\boldsymbol{n}}}_{{\boldsymbol{r}}}\times {\dot{{\boldsymbol{n}}}}_{{\boldsymbol{r}}},{{\boldsymbol{n}}}_{{\boldsymbol{r}}}\cdot {\partial }_{y}{{\boldsymbol{n}}}_{{\boldsymbol{r}}}\times {\dot{{\boldsymbol{n}}}}_{{\boldsymbol{r}}}\mathrm{).}$$

In the system without impurities *K*_imp_ = 0, skyrmion has no time-dependent distortion in the current driven motion and it is found that

5 $${E}_{x}=-\,{N}_{sk}{v}_{d,y},\,\,{E}_{y}={N}_{sk}{v}_{d,x},$$

where

6 $${N}_{sk}=\frac{1}{4\pi }\iint {{\boldsymbol{n}}}_{{\boldsymbol{r}}}\cdot {\partial }_{x}{{\boldsymbol{n}}}_{{\boldsymbol{r}}}\times {\partial }_{y}{{\boldsymbol{n}}}_{{\boldsymbol{r}}}{d}^{2}r$$

is the skyrmion number and ***v***_*d*_ = (*v*_*d*,*x*_, *v*_*d*,*y*_) is the velocity of the skyrmion. The total number of skyrmions is given by |*N*_*sk*_| and the single skyrmion magnetic texture {***n***_***r***_} results in *N*_*sk*_ = −1 in the present model.

The right panels of Fig. 2(a) show the time dependence of the quantities *E*_*x*_/*j* (blue line) and *E*_*y*_/*j* (red line). We find a correspondence between the skyrmion trajectory and the plots of *E*_*x*_/*j* and *E*_*y*_/*j* in Fig. 2(a): For *t* ≥ 65000(=*t*_*p*_), *E*_*y*_/*j* and *E*_*x*_/*j* show the damped oscillations around zero reflecting the pinned behavior of the skyrmion. For *t* ≤ *t*_*p*_, on the other hand, *E*_*y*_/*j* and *E*_*x*_/*j* largely fluctuate with large magnitude. From the trajectory of the skyrmion, we calculate the mean velocity of the skyrmion ${\bar{{\boldsymbol{v}}}}_{d}$ for *t* ≤ *t*_*p*_, and find ${\bar{{\boldsymbol{v}}}}_{d}/j=\mathrm{(1.079,}-\,\mathrm{0.165)}$. We also calculate ${\int }_{0}^{{t}_{p}}dt({\boldsymbol{E}}/j)/{t}_{p}$ and it gives (−0.165, −1.079) which is consistent with the relation Eq. (5). Even in the disordered system, ***E*** is available to discuss the dynamics of the skyrmion.

We see the pinned behavior for *j* ≤ 0.02, i.e., with decreasing *j*, the skyrmion travel-distance decreases and the skyrmion shows a damped oscillation nearby the initial position when *j* is small enough.

As discussed by previous study^41^, the current driven skyrmion shows the characteristic *breathing* dynamics. Here, during the traveling, we also see the breathing dynamics and spin wave emission (see also the supplementary movie for Fig. 2). The breathing amplitude becomes larger for larger *j*. This breathing eventually brings about a substantial *distortion* of the skyrmion and skyrmion multiplication discussed below.

With increasing the current density *j*, i.e., for 0.02 < *j* < 0.2, we find the multiplication of the skyrmion. Figure 2(b) shows a time evolution of the magnetic texture in the skyrmion multiplication for *j* = 0.1. Under this current density, the moving skyrmion is strongly distorted (see the magnetic texture at *t* = 75230 in Fig. 2(b)). In the present system, a skyrmion has the skyrmion nunber *N*_*sk*_ = −1. In the time-evolution, a part of the skyrmion is squeezed and eventually the skyrmion is separated into two magnetic textures, upper small one and lower elongated one, (see the magnetic texture at *t* = 75685 in Fig. 2(b)). This occurs within a continuous deformation of the magnetic texture, i.e., it is numerically confirmed that the total skyrmion number *N*_*sk*_ does not change along the time-evolution up to *t* = 75685. At *t* = 75685, the upper small magnetic texture has *N*_*sk*_ = −1 and is a distorted skyrmion (c.f., the skyrmion texture in Fig. 2(a)). The rest of the magnetic texture is a topologically *trivial* one, i.e., it has *N*_*sk*_ = 0: The arrows in the magnetic texture at *t* = 75685 in Fig. 2(b) indicate the winding texture of the in-plane magnetic moments of the upper end of the lower elongated magnetic texture. This winding texture is *not* compatible with the DM interaction and is reversed with a spin wave emission along the time-evolution for 75685 ≤ *t* ≤ 75795 (see Fig. 2(b)). At the same time, the skyrmion number changes. The resulting magnetic texture has *N*_*sk*_ = −2 in total, i.e., a multiplication of skyrmion occurs.

**Figure 3:**
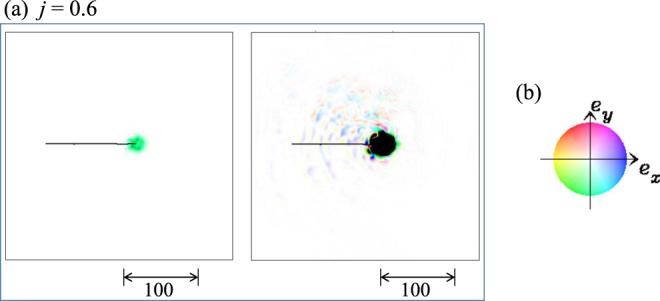
Spin wave creation/emission by current driven single skyrmion motion for {*α* = *β* = 0.01, *K*_imp_ = 0.2, *h* = 0.025, *j* = 0.6}. (**a**) Spatial distribution of emergent e-field ***e***_***r***_(*t*) at *t* = 200 using color code (**b**). Blue (yellow) means positive (negative) in *x* component of the e-field. The darkness represents the magnitude of the e-field, i.e., white is zero e-field. In the left (right) panel of (**a**), a cutoff 0.01 (0.0002) is introduced, so that the e-field larger than the cutoff magnitude is represented by black color.

With further increasing *j*, i.e., for the current density *j* ≥ 0.2, the skyrmion multiplication is not seen, and the distortion of the skyrmion becomes small. We find that the skyrmion moves almost along the current direction and the transverse velocity is very small due to the strong STT effect. In addition, we find a substantial spin wave creation/emission by current driven single skyrmion motion in the disordered system with the large current density. Figure 3 shows the time evolution of the emergent e-field for {*α* = *β* = 0.01, *K*_imp_ = 0.2, *h* = 0.025, *j* = 0.6} and the black line in Fig. 3(a) represents the trajectory of the skyrmion. (Note that the color code Fig. 3(b) is for ***e***_***r***_ not for ***n***_***r***_). Because the definition of the e-field Eq. (4) involves the time derivative of the magnetic moments ${\dot{{\boldsymbol{n}}}}_{{\boldsymbol{r}}}$, the spatial distribution and its time evolution give a measure of those of the spin wave(s). As seen in Fig. 3(b), the spatial distribution of the spin wave is not symmetric from the center of the skyrmion. In particular, Fig. 3 clearly shows the spin wave is created and emitted from the moving skyrmion and the large amplitude is concentrated along the trajectory of the skyrmion. It is confirmed that such spin wave creation/emission does not occur in the case that *K*_imp_ = 0, i.e., in the system without impurities.

### Current driven dynamics of many skyrmions

**Figure 4:**
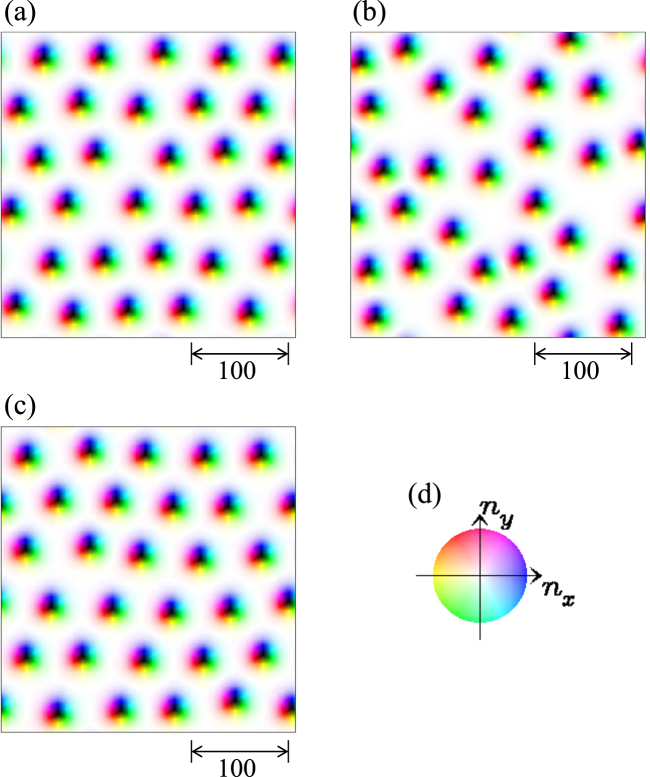
Current driven skyrmion dynamics with weak impurity strength. A parameter set {*h* = 0.025, *K*_imp_ = 0.01, *α* = *β* = 0.01} is used (see also Methods). (**a**) Magnetic texture at initial state. Magnetic textures at *t* = 200000 for (**b**) *j* = 0.001 and (**c**) *j* = 1.0 are shown. (**d**) Color code for in-plane magnetic moment.

In the absence of impurities *K*_imp_ = 0, the SkX phase is stabilized for *h* = 0.025, and the SkX magnetic texture is kept under the current driven motion. At *K*_imp_ = 0.01, we examine the current driven skyrmion dynamics for *j* ≥ 0.001 (see Fig. 4). Initial state shown in Fig. 4(a) is the relaxed SkX magnetic texture at *K*_imp_ = 0.01, and hence the skyrmion arrangement is slightly distorted from the purely triangular one. Figure 4(b) is the magnetic texture under the current *j* = 0.001 at *t* = 200000. In this case, the impurity effect is not strong enough to pin all the skyrmions, and the skyrmions show a moving gas like behavior, which is denoted SkG in Fig. 1. For larger *j*, the impurity effect becomes less important. Figure 4(c) shows a snapshot of the moving skyrmions for *j* = 1.0 at *t* = 200000. The (distorted) SkX arrangement seen in the initial state Fig. 4(a) is almost kept during the current driven motion in this case.

For larger *K*_imp_, we find the pinned state. The critical current density for *K*_imp_ = 0.2 is smaller than that for the single skyrmion discussed in Fig. 2. Namely, the skyrmions are moving for *j* ≥ 0.006 (see Fig. 1(a)) for many skyrmions although the single skyrmion shows the pinned behavior for the current density. This is because the skyrmions kick the other skyrmions before they are eventually pinned during the motion as shown in Fig. 2(a).

**Figure 5:**
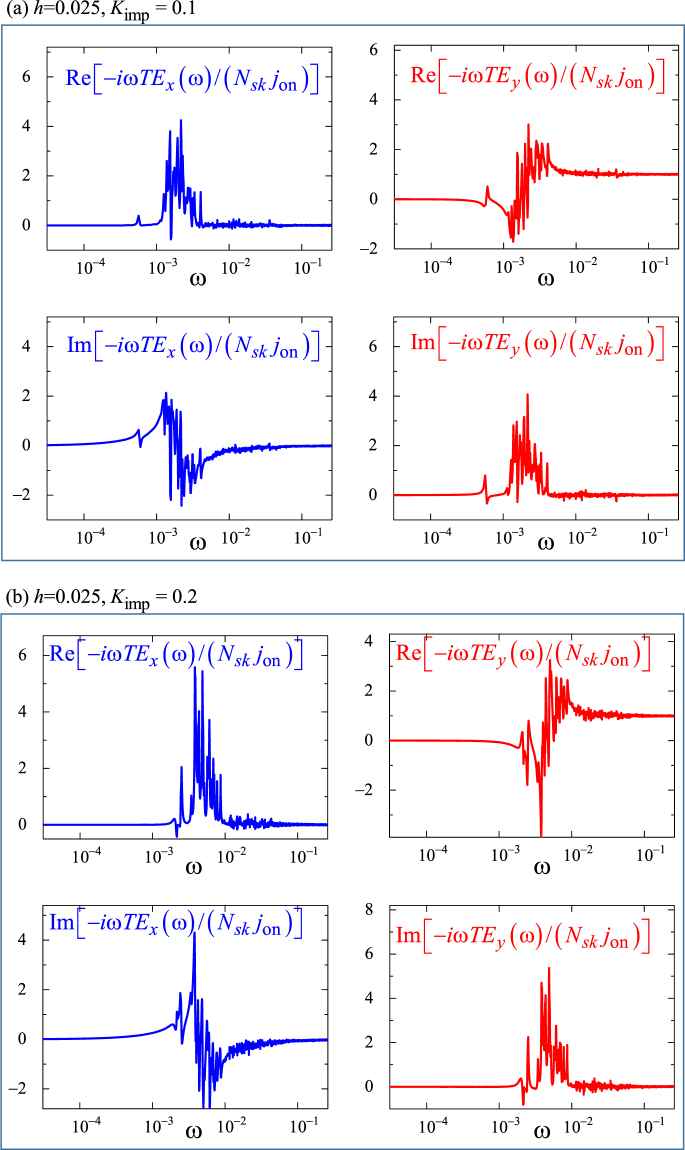
Fourier spectra of *E*_*x*_ and *E*_*y*_ for pinned state (see text). The *ω* dependences of the −*iωTE*_*x*_(*ω*)/(*N*_*sk*_*j*_on_) (blue) and −*iωTE*_*y*_(*ω*)/(*N*_*sk*_*j*_on_) (red) for {*h* = 0.025, *K*_imp_ = 0.1} and {*h* = 0.025, *K*_imp_ = 0.2} are shown in (**a**) and (**b**), respectively.

To examine the pinned SkX dynamics in more detail, we calculate the Fourier spectra of *E*_*x*_ and *E*_*y*_ (see Fig. 5): For *K*_imp_ = 0.2 with *h* = 0.025, we apply the current *j* = 0.001 (=*j*_on_). The initial state is the relaxed SkX state in the disordered system. At the first stage, every skyrmions show the damped oscillation nearby the initial positions without large travel-distances, and eventually, the oscillating behavior almost disappears at *t* = 200000 (=*t*_1_). In particular, the magnitudes of *E*_*x*_ and *E*_*y*_ at *t* = *t*_1_ are negligibly small. At *t* = *t*_1_, we remove the current, i.e., *j* = *j*_on_ → 0. Following this, the *pinned* skyrmions start the relaxation dynamics. In this relaxation dynamics, the skyrmions show again damped oscillation and the oscillating behavior almost disappears at *t* = 400000 (=*t*_2_). For this time interval *T* = *t*_2_ − *t*_1_, we calculate the Fourier spectra ${\boldsymbol{E}}(\omega )=\mathrm{(1/}T){\int }_{{t}_{1}}^{{t}_{2}}{\boldsymbol{E}}{e}^{-i\omega t}dt$ and the results is shown in Fig. 5. In the same way, the relaxation dynamics for *K*_imp_ = 0.1 is also examined. Note that this Fourier spectra give the linear response of skyrmions to the external current. Therefore, we expect the resonant behavior around the pinning frequency in analogy to the case of pinned charge density wave^47–51^. It is actually observed in Fig. 5, and the pinning frequency is of the order of $\omega \sim {10}^{3}$ in Fig. 5(a) with *K*_imp_ = 0.1, and $\omega \sim 4\times {10}^{-3}$ in Fig. 5(b) with *K*_imp_ = 0.2. Clearly it increases as *K*_imp_ increases.

During the relaxation dynamics in the time interval *T*, the time-dependent distortion of the each skyrmion is negligibly small. Therefore, the Thiele equation (see Methods) is available to understand the skyrmion dynamics. For $t > {t}_{2}$, *j* = 0, so that the Thiele equation gives the velocity of a skyrmion *v*_*d*,*x*_(*t*) ≈ −*F*_*y*_/(4*π*) and *v*_*d*,*y*_(*t*) ≈ *F*_*x*_/(4*π*) by the pinning force ***F*** = (*F*_*x*_, *F*_*y*_) for small *α* (=0.01 in the present case). Note that *E*_*y*_(*t*) = −*v*_*d*,*x*_(*t*) and *E*_*x*_(*t*) = *v*_*d*,*y*_(*t*) (Eq. (5) with *N*_*sk*_ = −1 for a skyrmion), and hence the relation

7 $$\{\begin{array}{l}{\rm{Re}}[-i\omega {E}_{x}(\omega )]\approx {\rm{Im}}[-i\omega {E}_{y}(\omega )]\\ {\rm{Im}}[-i\omega {E}_{x}(\omega )]\approx -{\rm{Re}}[-i\omega {E}_{y}(\omega )]\end{array}$$

is seen in Fig. 5 for the relaxation dynamics with tiny oscillation amplitude of the skyrmions at around the pinned skyrmion positions. At large *ω* limit, the impurity effect becomes less important. In the absence of pinning force, the Thiele equation gives

8 $$\{\begin{array}{rcl}{v}_{d,x}(t) & = & {j}_{{\rm{on}}}\mathrm{(1}-u(t-{t}_{1}))\\ {v}_{d,y}(t) & = & 0\end{array}$$

(*u*(*t*) is the unit step function) for *α* = *β* being the condition discussed here, and hence *v*_*d*,*x*_(*ω*) = *j*_on_/(−*iω*). This consequence Eq. (8) is seen as −*iωTE*_*y*_(*ω*)/(*N*_*sk*_ *j*_on_) → 1 and *E*_*x*_(*ω*) → 0 at the large *ω* limit in Fig. 5.

**Figure 6:**
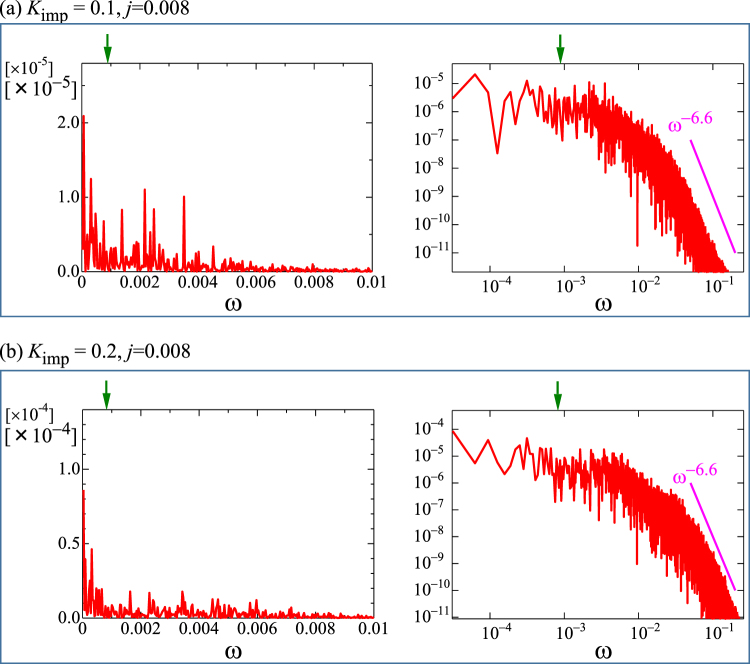
Fourier spectral weight of *E*_*y*_ for depinned state. A parameter set {*h* = 0.025, *j* = 0.008, *α* = *β* = 0.01} is used. (**a**) |*E*_*y*_(*ω*)|^2^ (red) as a function of *ω* for *K*_imp_ = 0.1. Green arrows indicate *ω*_*wf*_ (see text). (**b**) The same as (**a**), but *K*_imp_ = 0.2. Purple line is a guide for eyes to evaluate the slope of |*E*_*y*_(*ω*)|^2^ at large *ω*.

We also examine the power spectra |*E*_*y*_(*ω*)|^2^ as shown in Fig. 6 for the depinned skyrmion dynamics. For the current *j* = 0.008 at *K*_imp_ = 0.1 and 0.2, skyrmions flow under the impurity effect. This corresponds to the conduction noise of the moving skyrmions. The analogy to the charge density wave reminds us the sharp peak at the “washboard frequency” *ω*_*wf*_ = 2*πv*/*λ* with *v* = |***v***_*d*_| being the velocity of skyrmions and *λ* the period of the SkX state. In the present case, *ω*_*wf*_ will be given by $\lambda =\sqrt{L/|{N}_{sk}|}$ (*L*: system size) and *v* = |***E***(*ω* = 0)/*N*_*sk*_|, and it is indicated by green arrows in Fig. 6. However, not only at *ω* = *ω*_*wf*_, but also at whole *ω* region, we do not find characteristic peak structure in Fig. 6. This is because the particle nature of the skyrmions is more appropriate compared with the density wave picture. There are enough spaces between skyrmions, which behave more like individual particles, and hence there is no well-defined periodicity of the motion. In the log-log plot, |*E*_*y*_(*ω*)|^2^ shows weak *ω* dependence at small *ω* and at large *ω* it drops as $\sim {\omega }^{-6.6}$. This behavior is basically consistent with those observed by previous studies^35–38^.

**Figure 7:**
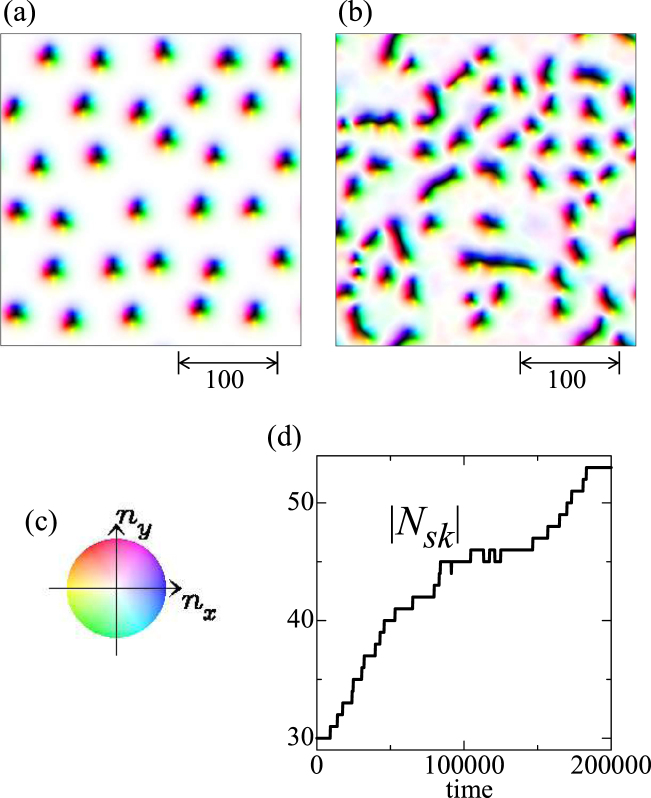
Skyrmion multiplication. A parameter set {*h* = 0.025, *K*_imp_ = 0.2, *α* = *β* = 0.01, *j* = 0.04} is used. (**a**) Magnetic texture at initial state. (**b**) Magnetic texture at *t* = 200000. (**c**) Color code for in-plane magnetic moment. (**d**) Time dependence of the total number of skyrmion |*N*_*sk*_|.

For *K*_imp_ = 0.2 and 0.02 < *j* < 0.4, we find the skyrmion multiplication (i.e, the dark blue region in Fig. 1(a)), similar to the single skyrmion dynamics shown in Fig. 2(b). Figure 7 shows an example of the skyrmion multiplication at the parameter set {*h* = 0.025, *K*_imp_ = 0.2, *α* = *β* = 0.01, *j* = 0.04}. Initial state (Fig. 7(a)) is the relaxed SkX state without current, *j* = 0, in this disordered system. Under the current *j* = 0.04, skyrmions flow by the STT effect, and due to the impurity effect, strong distortion and multiplication of the skyrmions occur and total number of skyrmions |*N*_*sk*_| increases by almost twice at *t* = 200000 (see Fig. 7(b) and (d)).

**Figure 8:**
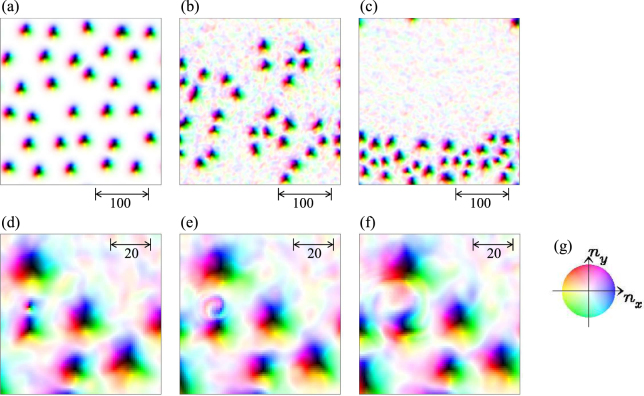
Skyrmion segregation. A parameter set {*h* = 0.025, *K*_imp_ = 0.2, *α* = *β* = 0.01, *j* = 0.6} is used. (**a**) Initial state. The snapshots at (**b**) *t* = 100000 and (**c**) *t* = 200000, (**d**) *t* = 241880, (**e**) *t* = 241887.5, (**f**) *t* = 241893.5 are also shown. (**g**) The color code for the magnetic texture.

With further increasing *j*, we find the segregation of the skyrmions. Figure 8 shows the skyrmion dynamics for *K*_imp_ = 0.2 and *j* = 0.6. The initial state (see Fig. 8(a)) is the relaxed SkX magnetic texture in this disordered system. Along the current driven skyrmion dynamics, the skyrmions show the stripe formation and finally, the skyrmion stripe along horizontal direction is formed. The important ingredient for the skyrmion segragation is the spin wave emission of the skyrmion discussed in Fig. 3. In the previous study^52^, it is shown that the spin wave attracts the skyrmion. Because the spatial distribution of the spin wave is not symmetric from the center of the skyrmion, the spin wave mediated attractive interaction between the skyrmions brings about the spatially anisotropic skyrmion stripe formation as seen in Fig. 8.

The skyrmion density inside the stripe becomes denser along the time-evolution, and eventually, some of skyrmions are crushed and the skyrmion number decreases. Figures 8(d–f) are the snapshots of the magnetic texture during the typical skyrmion annihilation process.

The skyrmion segregation occurs along the current driven motion, so that the skyrmion stripe is relaxed and the skyrmion density becomes sparse after the current is turned off, *j* = 0. It is well known that the distortion of magnetic structures associated with the motion leads to the inertia, i.e., the mass of the magnetic texture. Therefore, it is an interesting issue weather the mass for the center of mass motion is generated by the disorder for the skyrmions.

**Figure 9:**
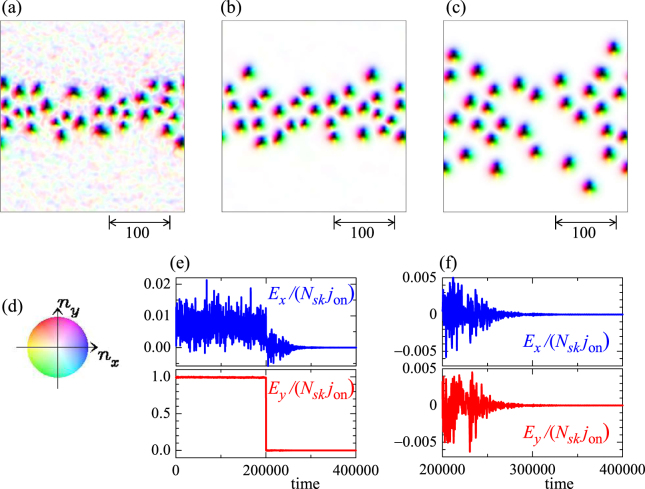
Shutoff of current *j* and skyrmion relaxation dynamics. (**a**) This magnetic texture is identical with Fig. 8(c), but the center of mass in *y* direction is shifted. Note that periodic boundary condition is applied. Here, at *t* = 200000, the current *j* is turned off, i.e., *j* = 0.6 (=*j*_on_) → 0. (**b**) The snapshot of magnetic texture at *t* = 204000. (**c**) The snapshot of magnetic texture at *t* = 400000. (**d**) Color code for the magnetic texture. (**e**) Time dependence of *E*_*x*_/(*N*_*sk*_ *j*_o*n*_) (blue) and *E*_*y*_/(*N*_*sk*_ *j*_o*n*_) (red). (**f**) The same as (**e**), but after the current *j* is turned off. Note the change in the scale.

Figure 9 shows the skyrmion relaxation dynamics after the current is turned off, i.e., *j* = 0.6(=*j*_on_) for 0 < *t* ≤ 200000 and *j* = 0 for 200000 < *t*. The magnetic texture shown in Fig. 9(a) is identical with Fig. 8(c), but the center of mass in *y* direction is shifted. (Note that periodic boundary condition is applied here). Figure 9(b) and (c) are the snapshots at *t* = 204000, and *t* = 400000, respectively. To examine the skyrmion velocity, the time dependences of *E*_*x*_ and *E*_*y*_ are also shown in Fig. 9(e) and (f). As discussed in Fig. 3, for the large *j*, due to the strong STT effect, the skyrmion trajectory shows the small deviation from the skyrmion motion in the absence of the impurities. Therefore, we find tiny *E*_*x*_ and *E*_*y*_/*j*_on_ ≈ *N*_*sk*_ for 0 < *t* ≤ 200000 and this large current density *j*_on_ = 0.6. At *t* = 200000, the current is turned off and at the same time, *E*_*y*_ is suddenly suppressed. The skyrmion dynamics in this disordered system appears as the damped oscillation of *E*_*x*_ and *E*_*y*_ with tiny amplitude, and finally all the skyrmions become silent at the trapped positions by the impurities. Therefore, there is no inertia effect for the center of mass motion of skyrmions although the relaxation occurs for the internal structure.

### Case of *h* = 0.04

**Figure 10:**
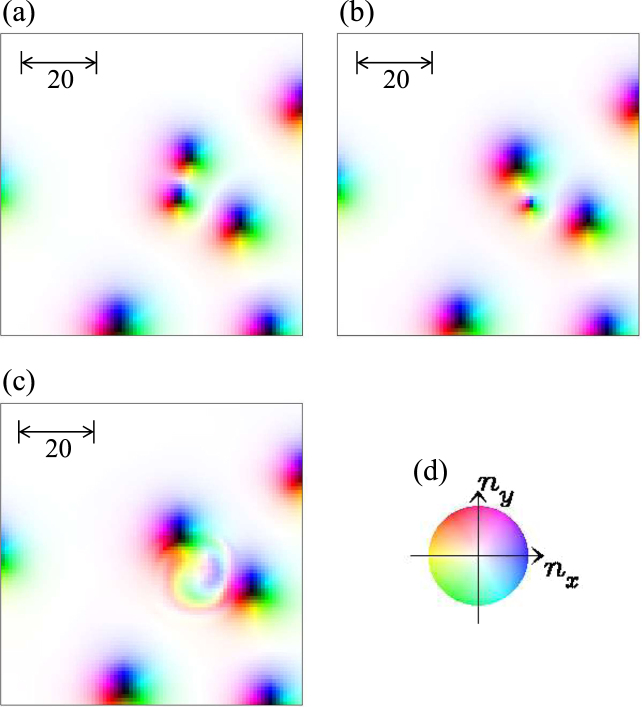
Skyrmion annihilation. A parameter set {*h* = 0.04, *K*_imp_ = 0.2, *j* = 0.01, *α* = *β* = 0.01} is used. The snapshots at (**a**) *t* = 25770 and (**b**) *t* = 25900, (**c**) *t* = 25920 are shown. (**d**) The color code for the magnetic texture.

We also examine the skyrmion dynamics for larger magnetic field *h*. For the parameter set {*J* = 1.0, *D* = 0.2, *h* = 0.04} corresponding to Fig. 1(b), the perfect ferromagnetic state is the ground state and the SkX state is the metastable state for *K*_imp_ = 0. As seen in Fig. 1(b), the current driven dynamics is essentially the same as the case in Fig. 1(a), i.e., for *h* = 0.025, except for absence of the skyrmion multiplication and moving SkX phase with larger *j* and (very) smaller *K*_imp_. The large magnetic field *h* makes the skyrmion size small. At the same time, the range of the repulsive interaction between skyrmions is also reduced. The SkX with such small repulsive interaction is easily distorted by the current driven motion in the disordered system. For *K*_imp_ = 0.2 and *j* ≥ 0.4, we find the clear skyrmion segregation behavior similar to that discussed above. In the skyrmion segregation, the annihilation of the skyrmion also occurs. However, the skyrmion annihilation occurs even for the smaller current density. The example for *j* = 0.01 is shown in Fig. 10. The clear contrast between the multiplying in Fig. 1(a) and decreasing in Fig. 1(b) of skyrmions in the intermediate current region corresponds to the stability of the skyrmions in the ground state without the current, i.e., skyrmions are stable at *h* = 0.025 (Fig. 1(a)) while they are metastable at *h* = 0.04 (Fig. 1(b)).

## Discussion

We have studied numerically the dynamics of skyrmions with disorder under the current. There are several unique features of this system compared with the other related systems. Vortices in type-II superconductors^42,43^ are the most similar system, which also have the chiral dynamics and are driven by the current. However, there are three essential differences. First, the magnetic flux density ***B*** cannot terminate or originate because of the absence of the magnetic monopoles and anti-monopoles. Therefore, vortices and ***B*** should be considered together as a three-dimensional system even when one treats the two-dimensional superconductor. In contrast, the emergent magnetic field ${b}_{{\boldsymbol{r}},{i}}=\frac{1}{2}{e}_{ijk}{{\boldsymbol{n}}}_{{\boldsymbol{r}}}\cdot ({\partial }_{j}{{\boldsymbol{n}}}_{{\boldsymbol{r}}}\times {\partial }_{k}{{\boldsymbol{n}}}_{{\boldsymbol{r}}})$ associated with the skyrmions can have monopoles and antimonopoles, which exist on surfaces in two-dimensional thin film. Furthermore, the magnetic charge is zero for a Bloch type skyrmion, and hence the dipolar interaction can be neglected there. Secondly, the Goldstone boson in superconductor is gapped due to the Higgs phenomenon, i.e., it is shifted up to be the conventional plasmon and cannot mediate the interaction between the vortices. In the case of chiral magnets, the spin wave excitation is in the low energy region even though gapped by the external magnetic field, which mediates the interaction between the skyrmions. Lastly, the dissipation for the vortex motion is usually large, and the inertia is irrelevant, i.e., overdamped motion. On the other hand, the Gilbert damping constant *α* is usually much smaller than unity, and the system is in the underdamped regime. To compare with the previous work on the dynamics of skyrmions with disorder, which regards each skyrmion with point particle and simulates the Thiele equation, we mention that the internal deformation of skyrmions, multiplication/annihilation of skyrmions, and also the exchange of spin wave between skyrmions, are essential for the strongly disordered case as discussed above. For the future studies, the extension of the present study to the three-dimensional case is an important issue. In that case, the skyrmion becomes a string, the end points of which are monopole and anti-monopole. The dynamics of this string and (anti)monopole in the presence of disorder will offer a rich physics including the manipulation of metastable states^53^.

## Methods

For the LLG simulation of the single skyrmion (many skyrmions) we use 300 × 300 (300 × 312) finite size system with periodic boundary condition. For the initial state, we first prepare the ground-state/metastable at *K*_imp_ = 0, and obtain the relaxed state. To obtain the phase diagram Fig. 1, we examine the skyrmion dynamics at the point (*K*_imp_, *j*) with {*K*_imp_ = 0.01, 0.1, 0.2} and {*j* = 0.001. 0.002, 0.004. 0.006, 0.008, 0.01, 0.02, 0.04, 0.06, 0.08, 0.1, 0.2, 0.4, 0.6, 0.8, 1, 0 }, and some other points. Most calculations are done within a time duration 200000, but at some points we try the LLG simulation for more longer time. For the single skyrmion motion, the skyrmion position ***r***_*sk*_ is identfied by ***r***_*sk*_ = ∑_***r***_[(−*N*_*sk*_ × *n*_*z*,***r***_ − 1) × ***r***]/∑_***r***_(−*N*_*sk*_ × *n*_*z*,***r***_ − 1).

For the impurity sites ***r***_i_ ∈ Λ (Λ: set of the random sites), we use the random-number generator developed by M. Matsumoto and T. Nishimura (http://www.math.sci.hiroshima-u.ac.jp/~m-mat/MT/emt.html). We have confirmed that the current-driven dynamics is semi-quantitatively similar for different impurity configurations, and hence the impurity average does not modify our conclusions in this paper.

For the single skyrmion, the Thiele equation is expressed to be,

9 $$4\pi {N}_{sk}\hat{{z}}\times (-{\boldsymbol{j}}-{{\boldsymbol{v}}}_{d})+\kappa (-\beta {\boldsymbol{j}}-\alpha {{\boldsymbol{v}}}_{d})+{\boldsymbol{F}}=\mathrm{0,}$$

where $\hat{{\boldsymbol{z}}}$ is the unit vector perpendicular to the plane forming the two dimensional system studied in this paper, ***j*** = (−*j*, 0) and the tensor *κ* is defined by *κ* = ∫*d*^2^*r*∂_*i*_***n***_***r***_∂_*j*_***n***_***r***_ (*i*, *j* = *x*, *y*).

## Electronic supplementary material

Supplementary material

Movie for Fig.2(a).

Movie for Fig.2(b).

Movie for Fig.3.

Movie for Fig.4(b).

Movie for Fig.4(c).

Movie for Fig.7.

Movie for Fig.8(a)->(b)->(c).

Movie for Fig.8(d)->(e)->(f).

Movie for Fig.9.

Movie for Fig.10.
