# Supplementary material for: Theory of current-driven skyrmions in disordered magnets
Source: Sci Rep. 2018 Apr 20;8:6328. doi: 10.1038/s41598-018-24693-5 (PMC5910446; doi:10.1038/s41598-018-24693-5)
Supplement: Supplementary file 1 — Supplementary material [file 41598_2018_24693_MOESM1_ESM.pdf]

Theory of current-driven skyrmions in disordered magnets

Supplementary material

Wataru Koshibae, Naoto Nagaosa

Fig2a.mp4:	Movie for Fig.2(a).
Fig2b.mp4:	Movie for Fig.2(b).
Fig3.mp4:	Movie for Fig.3.
Fig4b.mp4:	Movie for Fig.4(b).
Fig4c.mp4:	Movie for Fig.4(c).
Fig7.mp4:	Movie for Fig.7.
Fig8_1.mp4:	Movie for Fig.8(a)→(b)→(c).
Fig8_2.mp4:	Movie for Fig.8(d)→(e)→(f).
Fig9.mp4:	Movie for Fig.9.
Fig10.mp4:	Movie for Fig.10.
